# *Veillonella montpellierensis* Endocarditis

**DOI:** 10.3201/eid1107.041361

**Published:** 2005-07

**Authors:** Clarisse Rovery, Anne Etienne, Cédric Foucault, Pierre Berger, Philippe Brouqui

**Affiliations:** * Hôpital Nord, Marseille, France

**Keywords:** SARS-Cov, qRT-PCR, nucleocapsid, capture ELISA

## Abstract

*Veillonella* spp. rarely cause infections in humans. We report a case of *Veillonella* endocarditis documented by isolating a slow-growing, gram-negative microbe in blood cultures. This microbe was identified as the newly recognized species *Veillonella montpellierensis* (100% homology) by 16S RNA gene sequence analysis.

*Veillonella* are anaerobic, gram-negative cocci, part of the normal flora of the mouth, gastrointestinal tract, and vaginal tract. *Veillonella dispar*, *V. atypica*, and *V. parvula* have been cultured from human specimens. They are infrequently isolated in human infections. Rarely, *Veillonella* species have been the only etiologic agents identified in serious infections such as meningitis, osteomyelitis, prosthetic joint infection, pleuropulmonary infection, endocarditis, and bacteremia. A new species, *V. montpellierensis*, has recently been isolated from the gastric fluid of a newborn and from the amniotic fluid of 2 women (1). Its pathogenic role is still debated.

## The Study

A 75-year-old woman was admitted to the intensive care unit with septic shock. She had a history of diabetes mellitus. A cardiac murmur had been noted 8 years earlier but was not investigated further. On physical examination, the patient had aortic and mitral murmur. Reagent strip for urinalysis detected leukocytes and nitrites. After 3 blood cultures and urinalysis, the patient was treated for septic shock secondary to upper urinary tract infection with ceftriaxone, 2 g/day intravenously. The patient's condition rapidly improved with antimicrobial drugs and dopamine. Three days after admission, she was afebrile and hemodynamically stable; she was transferred to the urology department for acute pyelonephritis, which had not been confirmed by computed tomographic (CT) scan. Urine culture yielded *Gardnerella vaginalis*. Chest radiograph showed a patchy density of the right inferior pulmonary lobe confirmed by chest CT scan that suggested either pneumonia or neoplasia. On day 6, a transesophageal echocardiograph, performed because of the cardiac murmur, showed oscillating intracardiac masses on the aortic and mitral valves. Because the blood cultures were still negative, we determined that the patient had culture-negative endocarditis and replaced ceftriaxone with amoxicillin, 12 g/day for 6 weeks, in addition to gentamicin, 3 mg/kg/day for 3 weeks. On day 26, another transesophageal echocardiograph was performed and showed that the vegetation on the aortic valve had disappeared and the mitral vegetation was greatly reduced. The patient was discharged after 42 days of antimicrobial drug treatment, and follow-up was not possible.

On day 14 after sampling, 2 of 3 anaerobic blood cultures (automated blood culture BACTEC 9240 system (Becton Dickinson, Le Pont de Claix, France) yielded a slow-growing, gram-negative microbe. Blood was subcultured onto Columbia agar with 5% sheep blood (Mérieux, Marcy l'Etoile, France) under 5% CO_2_ and anaerobic atmosphere and resulted in small colonies. This slow-growing microbe was lost after 2 subcultures, and no isolate is available for further description. The isolate retrieved in the blood culture was identified by 16S rRNA gene sequence analysis. The template DNA was prepared from a few colonies that were isolated on the blood agar incubated anaerobically. DNA was extracted by using Fastprep DNA extraction kit (Ozyme, St Quentin en Yvelines, France) according to the manufacturer's recommendations and was subjected to polymerase chain reaction (PCR) targeting the 16S rRNA gene as previously described (2). Sequencing the PCR product (2) showed a 1,531-nucleotide sequence. This sequence shared 100% homology with that of *V. montpellierensis* (GenBank accession no. AY244769) and was already reported (GenBank accession no. AY244769) in a previous article (3). In this article, the isolated *Veillonella* strain (that was isolated from our patient) was first identified as "candidatus *V. atypica*" since the sequencing of the amplicon disclosed 94% sequence similarity with that of *V. atypica* (3). *V. montpellierensis* had not yet been described. PCR contamination was unlikely since this organism had never been amplified in our laboratory and negative controls remained negative.

## Conclusions

According to the modified Duke criteria (4), our patient had definite endocarditis. Anaerobic microbes do not commonly cause endocarditis (5). Most cases of anaerobic endocarditis are caused by anaerobic cocci, *Propionibacterium acnes*, and *Bacteroides fragilis* group (5). We describe the seventh reported case of well-documented infectious endocarditis in which a *Veillonella* species was the sole pathogen and the first due to *V. montpellierensis.* Characteristics of the 7 *Veillonella* endocarditis patients are summarized in the Table. Five of them fulfilled the Duke modified criteria for definite endocarditis; the 2 others were possible endocarditis. All previously reported cases of *Veillonella* endocarditis were due to either *V. dispar* (9,10), *V. parvula* (11), or *V. alcalescens* (6–8), currently considered *V. parvula* (12). One patient had no history of fever (7), and 1 patient had no preexisting valvular disease (8). Five patients had an infected mitral valve; 4 of the 5 had prosthetic valves. Our patient had mitral and aortic endocarditis. All patients had positive blood culture except 2, for whom the diagnosis was made by culturing the valve (6,11). *Veillonella* spp. had also been isolated from intravenous drug users with polymicrobial endocarditis (13); *V. parvula* was isolated from a lung abscess in a patient with echocardiographic vegetations, but blood cultures were negative (14). We could not test the susceptibility of the organism because the bacterium was lost on subculture. In treating infections with *Veillonella* species, penicillin has been the antimicrobial agent of choice (10). However, recent studies found a notably high resistance to penicillin G (MIC ≥2 μg/mL) (15). These penicillin G–resistant isolates showed generally reduced susceptibility to ampicillin or amoxicillin but remained susceptible to amoxicillin and clavulanate (15). We treated our patient for culture-negative endocarditis with amoxicillin. As the clinical state of our patient improved, we did not change antimicrobial agents.

**Table:** Summary of 7 reported patients with endocarditis due to Veillonella species*

*Veillonella* species isolated	Age	Sex	Infected valve	Preexisting valvular disease	Echo vegetation	Specimen site	Duration of illness before diagnosis	Treatment	Outcome
*V. alcalescens* (6)	51	Male	Prosthetic mitral	Y	Y	Blood†	–	Penicillin G (6 wk IV); surgery	Cured
*V. alcalescens* (7)	60	Male	Native aortic	Benign heart murmur	N	Valve‡	6 mo	Cephapirin and gentamicin (2 wk); oral penicillin V for 6 mo; surgery	Cured
*V. alcalescens* (8)	35	Male	Native mitral	N	–	Blood	7 mo	Sulfonamide, penicillin, heparin, and para-amino-hippurate (18 mo)	Cured
*V. dispar* (9)	56	Male	Prosthetic mitral	Y	Y	Blood	2 wk	Penicillin (6 wk)	Cured
*V. dispar* (10)	57	Female	Prosthetic mitral	Y	Y	Blood†	3 wk	Ampicillin (2 wk IV) and metronidazole (2 wk oral); oral clindamycin; surgery	Cured
*V. parvula* (11)	49	Male	Prosthetic mitral	Y	N	Valve‡	36 h	Metronidazole; surgery	Cured
*V. montpellierensis* (present work)			Native mitral and aortic	Y	Y	Blood	6 d	Ampicillin (6 wk) and gentamicin (3 wk)	Unknown

*Y, yes; N, no; echo, echocardiography. †Negative culture of valve specimens. ‡Negative culture of blood specimens.

Our isolate has recently been compared with 3 other isolates and classified as a new *Veillonella* species named *V. montpellierensis* by Jumas-Bilak et al. (1). We demonstrate here that *V. montpellierensis* is pathogenic for humans and may be included as a new agent of endocarditis caused by fastidious pathogens.

We report here the seventh case of endocarditis due to *Veillonella* spp. identified by PCR amplification and sequencing of 16S rDNA gene and the first case of endocarditis due to *V. montpellierensis*. This case reemphasizes the usefulness of molecular methods in identifying fastidious microorganisms and in describing new clinical entities (3).

## References

[1] Jumas-Bilak E, Carlier JP, Jean-Pierre H, Teyssier C, Gay B, Campos J. *Veillonella montpellierensis* sp. nov., a novel, anaerobic, gram-negative coccus isolated from clinical samples. Int J Syst Evol Microbiol. 2004;54:1311–6. 10.1099/ijs.0.02952-0 15280307

[2] Drancourt M, Bollet C, Carlioz A, Martelin R, Gayral JP, Raoult D. 16S ribosomal DNA sequence analysis of a large collection of environmental and clinical unidentifiable bacterial isolates. J Clin Microbiol. 2000;38:3623–30. 11015374 10.1128/jcm.38.10.3623-3630.2000 PMC87447

[3] Drancourt M, Berger P, Raoult D. Systematic 16S rRNA gene sequencing of atypical clinical isolates identified 27 new bacterial species associated with humans. J Clin Microbiol. 2004;42:2197–202. 10.1128/JCM.42.5.2197-2202.2004 15131188 PMC404640

[4] Li JS, Sexton DJ, Mick N, Nettles R, Fowler VGJ, Ryan T, Proposed modifications to the Duke criteria for the diagnosis of infective endocarditis. Clin Infect Dis. 2000;30:633–8. 10.1086/313753 10770721

[5] Brook I. Endocarditis due to anaerobic bacteria. Cardiology. 2002;98:1–5. 10.1159/000064684 12373039

[6] Zussa C, Ius P, Cesari F, Valfre C, Totis E, Canova C, Fortuitous detection of prosthetic valve endocarditis caused by an uncommon etiologic agent. J Thorac Cardiovasc Surg. 1994;107:1167–8. 8159046

[7] Greaves WL, Kaiser AB. Endocarditis due to *Veillonella alcalescens.* South Med J. 1984;77:1211–2. 10.1097/00007611-198409000-00048 6484702

[8] Loewe L, Rosenblatt P, Alture-Werber E. A refractory case of subacute bacterial endocarditis due to *Veillonella gazogenes* clinically arrested by a combination of penicillin, sodium paraaminohippurate, and heparin. Am Heart J. 1946;32:327–38. 10.1016/0002-8703(46)90793-4 20996761

[9] Houston SD, Taylor D, Rennie R. Prosthetic valve endocarditis due to *Veillonella dispar*: successful medical treatment following penicillin desensitization. Clin Infect Dis. 1997;24:1013–4. 10.1093/clinids/24.5.1013 9142818

[10] Loughrey AC, Chew EW. Endocarditis caused by *Veillonella dispar.* J Infect. 1990;21:319–21. 10.1016/0163-4453(90)94197-8 2273279

[11] Boo TW, Cryan B, O'Donnell A, Fahy G. Prosthetic valve endocarditis caused by *Veillonella parvula*. J Infect. 2005;50:81–3. 10.1016/j.jinf.2003.11.008 15603847

[12] Mays TD, Holdeman LV, Moore WEC, Rogosa M, Johnson JL. Taxonomy of the genus *Veillonella* Prévot. Int J Syst Evol Microbiol. 1982;32:28–36.

[13] Levine D, Hadley WK, Mills J. Sextuplibacterial endocarditis: a new world's record? South Med J. 1988;81:1592–3. 10.1097/00007611-198812000-00033 3201306

[14] Sanchez MP, Inchaustegui BL, Ruiz DL. Infective endocarditis and pulmonary abscess in an intravenous drug addict caused by *Veillonella parvula.* Rev Clin Esp. 1991;188:382–3. 1784772

[15] Nyfors S, Könönen E, Bryk A, Syrjänen R, Jousimies-Somer H. Age-related frequency of penicillin resistance of oral *Veillonella*. Diagn Microbiol Infect Dis. 2003;46:279–83. 10.1016/S0732-8893(03)00082-8 12944020

